# Complete Genome Sequence of *Escherichia coli* Strain M8, Isolated from *ob/ob* Mice

**DOI:** 10.1128/genomeA.00449-17

**Published:** 2017-06-01

**Authors:** Jay Siddharth, Mathieu Membrez, Anirikh Chakrabarti, Bertrand Betrisey, Chieh Jason Chou, Scott James Parkinson

**Affiliations:** Gastrointestinal Health and Microbiome, Nestlé Institute of Health Sciences, Lausanne, Vaud, Switzerland

## Abstract

*Escherichia coli* is one of the common inhabitants of the mammalian gastrointestinal track. We isolated a strain from an *ob/ob* mouse and performed whole-genome sequencing, which yielded a chromosome of ~5.1 Mb and three plasmids of ~160 kb, ~6 kb, and ~4 kb.

## GENOME ANNOUNCEMENT

*Escherichia coli* has been frequently isolated from stool samples (1, 2) and is used as an indicator of fecal contamination in water supplies (3). In recent years, it has also been found to be associated with chronic and acute infections manifesting in various disease conditions (4, 5). There have been uses of *E. coli* as a probiotic (6), but a majority of the studies have been focused on pathogenic strains. Moreover, genomic information about *E. coli* variants have been specifically studied in the context of inflammatory conditions of the gut (7, 8). Strain M8 was isolated from conventionally raised *ob/ob* C57BL/6J mice using Drigalski medium.

Isolate M8 tested positive for beta-galactosidase, lysine decarboxylase, and ornithine decarboxylase activity using the API 20E system. Additionally, it tested positive for indole production and production of acids by metabolizing several sugar sources (glucose, mannitol, sorbitol, rhamnose, saccharose, melibiose, and arabinose). Isolate M8 showed strong positive activity for alkaline phosphatase, leucine arylamidase, acid phosphatase, and beta-galactosidase and weak activity for valine arylamidase, trypsin, phosphohydrolase, and beta-glucosidase using the API ZYM system.

Biochemically, isolate M8 tested positive for utilization of glycerol, d-arabinose, l-arabinose, ribose, d-xylose, galactose, glucose, fructose, mannose, rhamnose, dulcitol, mannitol, sorbitol, *N*-acetyl glucosidase, maltose, lactose, melibiose, sucrose, trehalose, raffinose, gentiobiose, l-fucose, and gluconate using the API 50 CHE system (aerobic and anaerobic). Analysis using the GEN III OmniLog system matched isolate M8 to the *E. coli* species. FAME lipid analysis of the M8 lipids matched those of *Shigella sonnei* GC, subgroup B, with a SIM index of 0.698, indicating that M8 shares high DNA homology with *E. coli*. This is consistent with FAME-based identification for *E. coli* isolates. The total proteomics showed a high homology with *E. coli* with a category match of level A. The closest species identified in the database was *E. coli* DSM 1576, which is a fecal isolate.

Total DNA was extracted using Qiagen genomic DNA tips, and the DNA was first used to perform an optical mapping of the genome. The DNA was subsequently used to prepare long distance (LJD) libraries and a shotgun library, which were sequenced with MiSeq V2 chemistry. The resulting paired-end reads were assembled *de novo* using Newbler version 2.9, and the gaps were closed after manual inspection with GapClosure version 1.12-r2 (part of SOAP*de novo*) using reads derived from Sanger sequencing. The overall assembly was guided using the optical mapping data; the assembly resulted in a singular chromosome of approximately 5.1 Mb and three plasmids of approximately 160 kb, 6 kb, and 4 kb each. These sequences were annotated using the NCBI Prokaryotic Genome Annotation Pipeline (http://www.ncbi.nlm.nih.gov/genomes/static/Pipeline.html).

### Accession number(s).

The completed whole-genome data are available at NCBI GenBank under BioSample number SAMN06445833, with accession numbers CP019953, CP019954, CP019955, and CP019956 for the chromosome and three plasmids.

## References

[1] Cabral JPS 2010 Water microbiology. Bacterial pathogens and water. Int J Environ Res Publ Health 7:3657–3703. doi:10.3390/ijerph7103657. PMC2996186 21139855

[2] Croxen MA, Law RJ, Scholz R, Keeney KM, Wlodarska M, Finlay BB 2013 Recent advances in understanding enteric pathogenic *Escherichia coli*. Clin Microbiol Rev 26:822–880. doi:10.1128/CMR.00022-13. 24092857 PMC3811233

[3] Gomes TA, Elias WP, Scaletsky IC, Guth BE, Rodrigues JF, Piazza RM, Ferreira LC, Martinez MB 2016 Diarrheagenic *Escherichia coli*. Braz J Microbiol 47(suppl 1):3–30. doi:10.1016/j.bjm.2016.10.015. 27866935 PMC5156508

[4] Titilawo Y, Obi L, Okoh A 2015 Occurrence of virulence gene signatures associated with diarrhoeagenic and non-diarrhoeagenic pathovars of *Escherichia coli* isolates from some selected rivers in South-Western Nigeria. BMC Microbiol 15:204. doi:10.1186/s12866-015-0540-3. 26449767 PMC4599032

[5] Piérard D, De Greve H, Haesebrouck F, Mainil J 2012 O157:H7 and O104:H4 Vero/Shiga toxin-producing *Escherichia coli* outbreaks: respective role of cattle and humans. Vet Res 43:13. doi:10.1186/1297-9716-43-13. 22330148 PMC3305544

[6] Wassenaar TM 2016 Insights from 100 years of research with probiotic *E. coli*. Eur J Microbiol Immunol 6:147–161. doi:10.1556/1886.2016.00029. PMC5063008 27766164

[7] Xue Y, Zhang H, Wang H, Hu J, Du M, Zhu MJ 2014 Host inflammatory response inhibits *Escherichia coli* O157:H7 adhesion to gut epithelium through augmentation of mucin expression. Infect Immun 82:1921–1930. doi:10.1128/IAI.01589-13. 24566630 PMC3993425

[8] Ahmed SA, Awosika J, Baldwin C, Bishop-Lilly KA, Biswas B, Broomall S, Chain PS, Chertkov O, Chokoshvili O, Coyne S, Davenport K, Detter JC, Dorman W, Erkkila TH, Folster JP, Frey KG, George M, Gleasner C, Henry M, Hill KK, Hubbard K, Insalaco J, Johnson S, Kitzmiller A, Krepps M, Lo CC, Luu T, McNew LA, Minogue T, Munk CA, Osborne B, Patel M, Reitenga KG, Rosenzweig CN, Shea A, Shen X, Strockbine N, Tarr C, Teshima H, van Gieson E, Verratti K, Wolcott M, Xie G, Sozhamannan S, Gibbons HS, Threat Characterization Consortium 2012 Genomic comparison of *Escherichia coli* O104:H4 isolates from 2009 and 2011 reveals plasmid, and prophage heterogeneity, including shiga toxin encoding phage stx2. PLoS One 7:e48228. doi:10.1371/journal.pone.0048228. 23133618 PMC3486847

